# Students’ Perceptions of Learning Analytics for Mental Health Support: Qualitative Study

**DOI:** 10.2196/70327

**Published:** 2025-08-01

**Authors:** Aglaia Freccero, Miriam Onwunle, Jordan Elliott, Nathalie Podder, Julia Purrinos De Oliveira, Lindsay H Dewa

**Affiliations:** 1 Division of Psychiatry, Department of Brain Sciences, Imperial College London, 2nd Floor Commonwealth Building, Du Cane Road, London, W12 0NN, United Kingdom, 44 7551893250; 2 School of Public Health, Faculty of Medicine, Imperial College London, London, United Kingdom; 3 Institute of Security Science & Technology, Imperial College London, London, United Kingdom; 4 Department of Physics, Imperial College London, London, United Kingdom; 5 Department of Life Sciences, Faculty of Natural Sciences, Imperial College London, London, United Kingdom; 6 NIHR Biomedical Research Centre, Imperial College London, London, United Kingdom

**Keywords:** learning analytics, big data, e-learning, higher education, data mining, mental health, well-being

## Abstract

**Background:**

Poor mental health among higher education students is a global public health concern. Learning analytics, which involves collecting and analyzing big data to support learning, could detect changes in behavior, learning patterns, as well as mental health and well-being. This could help inform mental health interventions in university settings. However, research has yet to explore students’ perspectives on using learning analytics for mental health and well-being purposes.

**Objective:**

This study aimed to explore students’ perspectives on using learning analytics to support students’ mental health and well-being at university.

**Methods:**

Semistructured interviews were conducted online using Microsoft Teams between June and July 2023. Participants were identified through university student unions, social media, and snowball sampling. In total, 3 university students aged 20‐26 years joined our team and formed our student advisory group (SAG). They informed the design, analysis, and dissemination stages of the research cycle. Braun and Clarke’s approach guided our thematic analysis. Data were triangulated by comparing codes from 2 transcripts across 2 independent researchers over a 2-hour online meeting. A coding framework was cocreated with the SAG to code the remaining transcripts and ensure data saturation. Themes were finalized and presented in a thematic map during a 2-hour meeting with the SAG and 2 researchers.

**Results:**

In total, 15 participants were interviewed. We identified three main themes: (1) potential of learning analytics for mental health and well-being innovation, (2) student involvement in decision-making regarding learning analytics, and (3) integration of learning analytics with existing support. Despite being initially unaware, students recognized the potential of using learning analytics as a monitoring and early intervention tool to support university students’ mental health. However, students raised concerns regarding data reliability and identified several ethical issues, such as privacy and lack of transparency. They also expressed the need to be involved in decision-making regarding learning analytics design, practices, and policies. Overall, students welcomed the possible integration of learning analytics with the existing university support.

**Conclusions:**

This is the first qualitative study to explore students’ perceptions of using learning analytics to support student mental health and well-being. Students’ generally positive attitudes toward learning analytics suggest that this tool could be effectively integrated into the existing university support systems. Considering the ethical concerns raised by students, our findings suggest the need to bring the student voice into learning analytics development and implementation.

## Introduction

Higher education students’ mental health is worsening in the United Kingdom and is an increasing public health and policy concern. Students are particularly vulnerable to poor mental health due to academic, financial, and psychosocial pressures [1]. In a recent survey by the charity Student Minds, 57% of students reported experiencing a current mental health problem, and 27% had a diagnosed mental health condition, the most common being depression and anxiety [2]. Over the past 10 years, the number of students who disclosed a mental health condition to their institution has increased almost fivefold [3], reflecting rising need, improved awareness, reduced stigma, and clearer reporting pathways. Poor mental health can result in poor academic performance, study discontinuation, and even death by suicide [3]. However, universities struggle to meet the growing demand for mental health support, highlighting the need to adopt mental health as a priority [4]. Innovative solutions are required to effectively address this crisis while meeting students’ mental health needs.

The integration of digital technology in higher education offers the opportunity to use students’ data to detect signs of deteriorating mental health and provide targeted interventions [5,6]. Learning analytics refers to the collection, analysis, and reporting of large datasets (often referred to as “big data”) of student data to understand and support student learning [7]. Learning analytics systems draw on data from various university systems, including student information systems, virtual learning environments, library usage, and attendance records [8,9]. While there is compelling evidence that learning analytics interventions can improve student outcomes (eg, retention, academic performance, and engagement) [10], there is limited research on their impact on mental health and well-being [11-13]. Changes in learning behavior may indicate changes in student mental health, although the current evidence remains scarce [14-16].

Few qualitative studies have explored students’ perceptions of learning analytics to support learning [17-25]. There is also a lack of research across different academic disciplines and education levels [17,19,20]. Only 3 studies focused on more than 1 institution [17-19], and the only study conducted in the United Kingdom used a small sample of final-year students from a single discipline [21]. Overall, these studies report that students generally welcome the use of learning analytics to enhance their educational experience [17-21], but also raise concerns about possible privacy breaches, lack of consent, equity issues, and poor data reliability [22-26].

Learning analytics has the potential not only to improve academic outcomes but also to identify and address students’ mental health and well-being needs in order to provide better support services [11-13]. Nonetheless, students are often not actively involved in decision-making regarding their implementation [4]. Understanding students’ views and engaging them in consultation is essential to inform learning analytics practices and the acceptable adoption of learning analytics for mental health purposes in higher education [15,16,27-30]. However, no studies have explored university students’ perceptions of using learning analytics for mental health and well-being. This study aims to fill this gap by exploring students’ perspectives on using learning analytics to support students’ mental health and well-being in university settings.

## Methods

### Study Design and Methodological Orientation

The study followed the Consolidated Criteria for Reporting Qualitative Research (COREQ) checklist (Checklist 1).

This study used a qualitative methodology to explore a “how” research question, focusing on the depth and complexity of an underresearched topic. Guided by a relativist ontology, subjectivist epistemology, and an interpretivist paradigm [31], our approach is well-suited to research in psychology, mental health, and education, where lived experience and sociocultural context are central [32,33]. A grounded theory methodology supported the inductive development of insights from participants’ perspectives [34]. Semistructured interviews were used to gather rich, nuanced data, aligned with the study’s exploratory aims [35].

### Participant Selection

Participants were identified through student networks, social media platforms (Instagram [Meta], X, and LinkedIn), and snowball sampling, an approach well-suited for exploring sensitive topics and effective in reaching diverse student populations, including international students [32,33]. Interested participants were encouraged to email the researcher to express their interest. Participants were eligible to take part if they were students (aged 18‐25 y) enrolled in an undergraduate or postgraduate course at a university in London, fluent English speakers, and willing to provide informed written consent. The age range represented students enrolled in undergraduate or postgraduate taught programs, which typically involve more structured online learning. Efforts were made to include diversity in gender, ethnicity, sexual orientation, degree, and year of study. All individuals who expressed interest and were contacted by the researcher met the eligibility criteria; none declined to participate or dropped out of the study. Participation was voluntary, and as this study was part of a master’s thesis, no compensation was provided.

### Data Generation

Participants’ demographic data were collected at recruitment (Table 1). Semistructured interviews were conducted by the lead researcher (AF) via Microsoft Teams between June 6 and July 27, 2023. Open-ended questions explored participants’ knowledge, attitudes, and concerns regarding the use of learning analytics to support university students’ mental health and well-being. The semistructured interview questions were informed by a review of literature on student mental health, learning analytics, and help-seeking in higher education. They were also guided by the technology acceptance model [36] to explore perceptions of learning analytics and self-determination theory [37] to consider factors influencing student motivation and engagement. Furthermore, 3 university students informed the topic guide (refer to Patient and Public Involvement section). It consisted of five sections: (1) introductory questions to help participants feel comfortable and at ease, (2) existing mental health support at university, (3) use of learning analytics to support university students’ mental health and well-being, (4) facilitators and barriers to learning analytics adoption for mental health and well-being purposes, and (5) integration of learning analytics within existing university mental health support services (Appendix S1 in Multimedia Appendix 1). All interviews were audio- and video-recorded, then transcribed verbatim. No one else was present during the interview except for the participants and the researcher. Transcripts were accessible to participants through Microsoft Teams. Field notes were taken during and after the interview and were considered when coding the transcripts.

**Table 1.** Participants’ demographics.

Characteristics	Value
Age (y), mean (SD)	21.47 (1.60)
Gender, n (%)	
Female	10 (67)
Male	5 (33)
Other	0 (0)
Ethnicity, n (%)	
White^a^	5 (33)
Asian or Asian British^b^	5 (33)
Mixed^c^	3 (20)
Black or Black British^d^	1 (7)
Other^e^	1 (7)
Qualification, n (%)	
Undergraduate	11 (73)
Postgraduate	4 (27)
Fee status, n (%)	
Home^f^	8 (53)
International^g^	7 (47)

a White: English, Welsh, Scottish, Northern Irish, British, Irish, Gypsy or Irish Traveller, Roma, or any other White background.

b Asian or Asian British: Indian, Pakistani, Bangladeshi, Chinese, or any other Asian background.

c Mixed or multiple ethnic groups: White and Black Caribbean, White and Black African, White and Asian, or any other mixed or multiple ethnic group.

d Black or Black British: Caribbean, African, or any other Black, Black British, or Caribbean background.

e Other ethnic group: Arab or any other ethnic group.

f Home fee status: students ordinarily resident or settled in the United Kingdom.

g International fee status: overseas or international students not subject to home fee status.

### Patient and Public Involvement

We advertised for individuals with lived experience (ie, university students whose data were being used by the university) through the Imperial College Union Mental Health Network Instagram account to join our team as advisors and inform the project. As a result, 3 students, aged 21‐26 years (2 female and 1 male), from the Faculty of Natural Sciences and the Faculty of Medicine at Imperial College London expressed interest and joined the team to form a student advisory group (SAG). The SAG included 1 postgraduate and 2 undergraduate students and informed 3 key stages of the research cycle [38]: designing and managing research, carrying out the research study, and dissemination.

We held a 2-hour online meeting (Microsoft Teams) on April 7, 2023, where the SAG informed the research objectives and accessibility and appropriateness of project documentation (interview topic guide, participant information sheet, consent form, and protocol). The following changes were made to the topic guide: language was simplified (ie, a lay definition of learning analytics was agreed upon), and a question was added regarding the impact of learning analytics on academic outcomes. The final topic guide was piloted with the SAG in 3 mock interviews. After providing all positive feedback, no subsequent changes were made to the topic guide. Following the generation of initial codes, a coding framework was cocreated with the SAG during an additional 2-hour online meeting (Microsoft Teams) on July 14, 2023. We held a final meeting on August 2, 2023, where the SAG and the 2 researchers (AF and MO) refined and consolidated themes. Several themes were revised, consolidated, and more effectively linked through student advisor involvement (Figure S1 in Multimedia Appendix 1). The SAG was not compensated for their time, as no budget was associated with this study, being a master’s thesis. Patient and public involvement (PPI) was reported using the Guidance for Reporting Involvement of Patients and the Public, Version 2-short version [39] (Table S1 in Multimedia Appendix 1).

### Data Analysis

An inductive and deductive method to Braun and Clarke’s 6-stage thematic analysis guided our analysis [40]. We used a data-driven approach to theme development while also drawing from the researcher’s previous knowledge of learning analytics. Transcripts were imported into Microsoft Word. After transcription, the lead researcher (AF) familiarized herself with the data by reading each transcript multiple times. All transcripts were manually coded in Microsoft Word, and relevant codes were compiled. Manual coding was chosen as it supported reflexivity, the development of the lead researcher’s skills, and enabled deep immersion in the data [41-43]. Furthermore, 2 independent researchers (AF and MO) compared codes from 2 transcripts during a 2-hour online meeting on Microsoft Teams. Data triangulation helped ensure the reliability and validity of the findings by identifying potential individual researcher biases and confirming the consistency of information. The coding framework was applied to code the remaining transcripts. Interviews were conducted until data saturation was reached, assessed through concurrent data analysis, so that no new codes emerged, and responses across participants consistently reinforced existing patterns. AF then organized codes into potential themes and subthemes and gathered all data relevant to each of these. Some themes were combined to avoid redundancy, while others were broken down into separate themes and judged for internal homogeneity and external heterogeneity. The thematic map was finalized with the SAG.

### Ethical Considerations

All procedures involving human participants were approved by the Head of Department of the School of Public Health and the Research Governance and Integrity Team at Imperial College London (Imperial College Research Ethics Committee reference 6604462). The authors assert that all procedures contributing to this work comply with the ethical standards of the relevant national and institutional committees on human experimentation and with the Helsinki Declaration of 1975, as revised in 2008.

Participants provided informed consent by signing and returning a consent form via email. A copy co-signed by both the participant and the researcher was emailed back to the participant and stored securely on Imperial College London encrypted drives accessible only to the study team. Participants could withdraw at any time before the start of data analysis. Audio and video recordings were deleted after transcription, and study-specific ID numbers were used to maintain participants’ anonymity. The principal investigator ensured confidentiality and General Data Protection Regulation compliance for health and care research. Participation was voluntary and uncompensated, as the study formed part of a master’s thesis.

## Results

### Participants’ Characteristics

In total, 15 participants took part in the study (10 female, 5 male), with a mean age of 21.47 (SD 1.60, range 19‐24) years (Table 1). Students from 4 London universities participated in the interview (Table S2 in Multimedia Appendix 1). Most participants were undergraduate students (11/15, 73%). All students were enrolled in a science, technology, engineering, and mathematics (STEM) degree. Interviews lasted between 19 and 34 minutes.

### Themes and Subthemes

**Figure 1.**
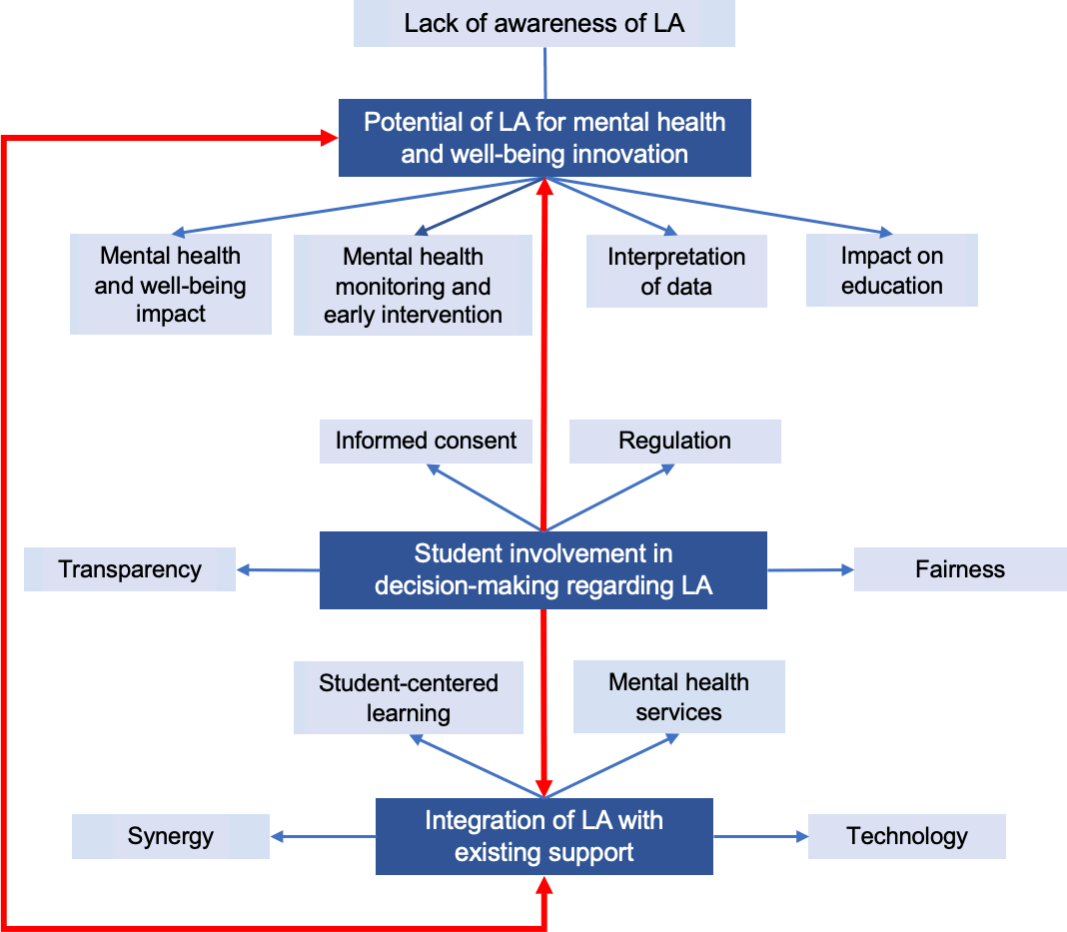
Thematic map displaying themes (dark blue), subthemes (light blue), and their relationships. Red arrows represent relationships between themes. Blue arrows represent the relationships between themes and their corresponding subthemes. LA: learning analytics.

There were three themes: (1) potential of learning analytics for mental health and well-being innovation, (2) student involvement in decision-making regarding learning analytics, and (3) integration of learning analytics with existing support (Figure 1).

#### Potential of Learning Analytics for Mental Health and Well-Being Innovation

At the beginning of the interview, all participants were generally unaware of learning analytics. While they were confident that their university had collected their data, such as their grades and feedback, most participants were unsure how these data were used. After providing them with an accurate definition of learning analytics (Appendix B in Multimedia Appendix 1), all participants felt more confident in their understanding of learning analytics and then recognized the potential impact of learning analytics to go beyond academic purposes.

*I think it has potential […] being used as an early indicator to intervene with mental health issues. I think when used correctly, you could potentially help students get the support they need.*

[ID10, female]

Specifically, they indicated learning analytics might serve as a tool for monitoring students’ mental health and well-being and informing appropriate intervention. Some students said that learning data collected by learning analytics could flag disengagement with studies, which might indicate a worsening in mental health.

*So let’s say you’ve never turned up to a lecture, that’s a red flag and that can be used by the mental health services… to intervene.*

[ID13, female]

They also highlighted the potential of learning analytics to serve as an early warning system for mental health issues and inform early intervention. Suggested interventions included automated messages via email or virtual learning environments, as well as direct contact from staff, such as mental health support teams and academic tutors, to check in with students in case of disengagement from studies and to signpost them to appropriate support.

*If someone is not attending because they are not doing well, having an email from a welfare perspective…may be a way to intervene a little bit earlier.*

[ID15, female]

Most students agreed that higher engagement with studies generally suggests better academic performance and that mental health and academic success are closely interconnected. They recognized that mental health difficulties could negatively impact academic engagement and performance, and that this underscored the need for learning analytics to consider these links when supporting students.

*It (learning analytics) would improve academic outcomes like through the well-being route […] because you know there’s no good academic outcome without good mental health.*

[ID4, female]

However, some students were skeptical about whether changes in learning behavior truly reflect changes in mental health. They reported concerns about the difficulty of interpreting data and how students have varied learning behaviors and mental health symptoms manifest differently from person to person.

*I can see why from that sort of behavioural data you can make conclusions about the learning…I’m just not sure how representative that is of one’s mental health.*

[ID14, female]

For instance, 1 participant suggested that some students might disengage from their studies when struggling, while others may immerse themselves in work as a coping mechanism. A few participants emphasized the importance of contextualizing behavioral changes and personalizing learning analytics to account for individual differences.

*With mental health…you can’t be definite. It’s so broad between individuals and not every mental health issue presents the same.*

[ID10, female]

#### Student Involvement in Decision-Making Regarding Learning Analytics

Most participants emphasized the need for clear, accessible, and transparent communication with students regarding the use of learning analytics for mental health and well-being. Many students reported that they should be made fully aware of what data will be collected, who will have access, where it will be stored, and how it will be used.

*Making it clear…why and what (data) is being collected, and that’s for your benefit.*

[ID8, male]

Some participants said better transparency would allow students to build trust in learning analytics, understand its benefits, and increase engagement with learning analytics systems.

*There needs to be transparency between the university and the students, otherwise…you can get to a point where students don’t necessarily trust the university*.

[ID13, female]

Importantly, many students stressed the importance of involving students as stakeholders in decision-making regarding the future development and implementation of learning analytics to ensure that learning analytics systems accurately reflect the realities of students’ mental health experiences.

*The thing I’m going to stress is involving the relevant stakeholders in deciding who’s going to have this data. How is it going to be used? What intervention, so what are you going to do when you identify these trends? Is that something that would be considered acceptable for students and staff and would it work? Would it also be helpful for those who find it acceptable?*

[ID15, female]

One student mentioned this involvement could mean coproducing learning analytics systems between students and university staff. Finally, 1 participant believed implementing learning analytics would require continuous review and improvement based on student feedback to maximize its benefits for mental health and better meet the needs of students.

However, some students highlighted barriers to learning analytics adoption, including the potential hesitancy among students to give informed consent due to privacy concerns.

*Probably the main issue would be students not wanting to consent based on privacy*.

[ID6, female]

A few students mentioned that consent is usually given without the students’ full understanding and approval. Consent is often buried in long documents that students rarely read, leaving them uneasy about how their data are being used. A few suggested offering opt-in and opt-out options to maintain freedom of choice regarding data sharing. Ethical and regulatory issues were also reported. Some students voiced worries about learning analytics being used as a tool for inappropriate monitoring, which could lead to potential privacy breaches and invasive interventions.

*If some people have issues with being monitored, … the data processing and being spied on…they might find it actually uncomfortable… and maybe even a little bit threatened.*

[ID1, female]

In addition, a few students reported that learning analytics interventions could come across as accusatory and might unfairly impact students’ grades, for example, if low engagement were to be detected by learning analytics.

*…as long is not held against me like “ohh this will impact you…on your grades...”*

[ID8, male]

Therefore, they expressed the desire for learning analytics to be implemented equitably for all students, despite their level of engagement and academic performance, ensuring that the support learning analytics might offer is uniform and nondiscriminatory.

#### Integration of Learning Analytics With Existing Support

A common issue expressed by several participants was the lack of motivation for students to engage with learning analytics, such as attendance tracking or feedback. However, a few participants suggested monetary incentives to help engage students with learning analytics. Engagement was regarded to be dependent on the perceived relevance and ease of use of learning analytics systems. As such, several participants noted that learning analytics must be effortlessly integrated with existing academic practices.

*I think take the amount of effort away from the students as much as possible, or else they won't want to participate.*

[ID5, male]

For example, many students reported that engagement with learning analytics could be useful in assessing students’ learning journeys and responses to workload or assessments.

*…they can figure out which way is best for students’ learning, and they could then be able to personalise that a bit more.*

[ID13, female]

Therefore, some believed it would help inform how to adapt teaching practices to improve students’ academic outcomes and experiences, which could motivate students to engage with learning analytics.

*As college knows at which period students feel most stressed, they can change their teaching objectives and make students feel less stressed.*

[ID12, female]

Many students believed that learning analytics could be integrated not only within existing academic practices but also with existing mental health support. Therefore, learning analytics could be an innovative tool to link academic and welfare services, providing more holistic support for students.

*Academics and welfare are really closely linked and actually…you asked me….what would I suggest as improvements, and one more thing to add there is integrating these two services. So why is welfare completely separate?*

[ID15, female]

However, some highlighted concerns about implementing this integration. For example, some felt that implementing learning analytics to link services would require time, resources, and service restructuring.

*I feel like in theory it should help improve it [mental health]. But I guess it just depends how they use them.*

[ID11, male]

Many students identified artificial intelligence (AI) as a potential solution for tracking students’ mental health and implementing timely interventions with minimal effort from academic staff. For example, they suggested that algorithms adjusted to individual differences could offer personalized recommendations to maximize mental health. However, some participants expressed skepticism about the accuracy of these automated systems and that they would be overly intrusive in their lives.

*Maybe just an AI system that keeps updating what their baseline should be and so any deviation from that baseline of normal someone could kind of alert a member of staff.*

[ID4, female]

## Discussion

### Key Findings and Comparison With Previous Work

To the authors’ knowledge, this is the first study to explore students’ perceptions of learning analytics to support students’ mental health and well-being. There were three main themes: (1) potential of learning analytics for mental health and well-being innovation, (2) student involvement in decision-making regarding learning analytics, and (3) integration of learning analytics with existing support.

All participants supported the use of learning analytics as a tool to support students’ mental health and well-being. The majority of participants felt that learning analytics could enhance access to and delivery of support services, advocating for an evidence-based and student-centered approach, in line with existing qualitative literature and policy recommendations on improving student mental health support [35-38]. However, some students also recognized potential challenges in using learning analytics to detect and intervene in case of student mental health deterioration. Overall, our findings show that students’ positive attitudes and acceptability of learning analytics hold promise for implementation within current university support systems. Learning analytics may help enhance coordination between academic and mental health services, supporting UK policy recommendations to adopt a whole-university approach to mental health [4].

Despite an initial lack of awareness of learning analytics, all students recognized its potential beyond the learning context to support mental health and well-being. This aligns with qualitative literature that reports students’ generally positive attitudes toward learning analytics [17,25] and digital tools for mental health monitoring and early intervention in young people [44-46]. Most participants believed that learning analytics could provide objective and reliable evidence for monitoring students’ mental health. They also welcomed its potential to inform early interventions and integrate with existing university support systems. However, they also recognized the potential challenges of using learning analytics for mental health purposes and stressed the need to be involved in decision-making about learning analytics development and implementation.

Several barriers and facilitators were identified for adopting learning analytics to support students’ mental health and well-being. Some students raised concerns regarding the reliability of learning data and identified several ethical issues, in line with other qualitative studies [14,17,18,25]. Students worried that data inaccuracy could lead to delivering inappropriate interventions, highlighting the need for a context-specific understanding of students’ behavior. Students also reported the importance of balancing the need to share data for effective interventions with concerns about privacy breaches and ensuring informed consent, reflecting findings from other studies [20,26,47-49] and aligning with proposed recommendations for ethics and privacy in learning analytics [50,51]. Participants agreed these risks might subside if universities were more transparent about using learning analytics and abided by good practice guidelines [14,15,28,30].

Overall, students’ ethical concerns underscore the need to adopt a coproduced approach in the development, implementation, and governance of learning analytics that strategically incorporates the student voice and aligns with the principles of PPI [15,16]. Meaningfully engaging students as active stakeholders, beyond their role as data subjects, is essential to fostering trust and ensuring that learning analytics systems are transparent, ethical, and responsive to student needs [27-30]. Regarding facilitators for learning analytics adoption, students stressed the need for incentives to engage with learning analytics, as well as staff inclusion and training, in line with other studies [19,21,23,52]. Our results support previous research [17,26], showing that learning analytics can be used to assess students’ learning methods and adjust teaching approaches and curricula to better support their mental health and academic needs. AI-driven computational approaches, including machine learning, natural language processing, and rule-based systems, were viewed positively. These methods were considered valuable additions to learning analytics systems, offering predictive capabilities and data-informed recommendations to enhance student learning and support students’ mental health when implemented with appropriate student involvement, as highlighted in a recent systematic review [53]. This aligns with qualitative research reporting young people’s acceptance of AI [54]. Therefore, our study suggests that students’ positive views on learning analytics support the potential for integrating learning analytics with existing academic and mental health support, as per UK policy recommendations [4].

### Reflexivity

The researcher’s perspective was informed by her lived experience as a student with mental health issues (Appendix S2 in Multimedia Appendix 1). This insider positionality facilitated access, built rapport, and enabled the development of informed, contextually relevant questions. However, it also introduced potential for unconscious bias, including overidentification with participants and possible influence from pre-existing views on university mental health provision. Furthermore, the researcher’s involvement in mental health advocacy within her institution may have led participants to have some awareness of her motivations for conducting the research.

To address these biases, a reflexive approach was used throughout the study to critically examine how personal assumptions, values, and previous knowledge might influence design, data collection, and interpretation [55,56]. Attention to the insider-outsider dynamic, reflexive journaling, PPI, and data triangulation with other researchers was used to ensure transparency and analytical rigor, grounding the findings in participants’ perspectives rather than the researcher’s personal views.

### Strengths and Limitations

There are several strengths to this study. A total of 3 people with lived experience and interest in mental health research informed the design, analysis, and interpretation research stages [57]. Students had personal knowledge and experience of mental health support at university, which made the research more relevant, focused, and accessible.

There are also study limitations. Despite efforts to maximize variation, participants’ backgrounds were not diverse in terms of gender and sexual orientation. Our sample was predominantly female, and we did not include individuals who did not identify with the gender assigned at birth. We also did not explore differences in sexual orientation, and our sample was predominantly heterosexual. LGBTQ+ students are particularly vulnerable to mental health problems [58], and their views would enrich the field. While the sample included both home and international students from a range of cultural and ethnic backgrounds, the study did not explicitly explore how cultural factors shape mental health experiences or help-seeking behaviors [59]. Furthermore, although participants came from multiple London universities, they were all STEM students, so our results may not be generalizable to other universities in the United Kingdom or students from other educational backgrounds. Future research could incorporate focus groups to explore the topic in a more naturalistic setting, thereby validating findings and minimizing researcher bias. Larger studies are also needed to enhance generalizability. Quantitative approaches, such as exploratory factor analysis, could be used to examine broader patterns based on the themes identified in this study.

### Implications and Policy Recommendations

This research focused on students’ perceptions of learning analytics to support students’ mental health and well-being. To ensure the successful adoption of learning analytics systems to support students’ mental health and well-being in higher education in the future, there is now a need to explore the perceptions of all other stakeholders, including academic staff, counseling and disability services, and IT services [60]. Our qualitative findings could also be complemented with a national survey across all UK universities and internationally to capture broader perspectives on the interview themes and explore relationships between the data. As learning analytics systems are implemented, future research should investigate the feasibility, acceptability, and effectiveness of these systems in improving students’ mental health and well-being.

There are also several practical and policy implications to this study. First, students’ general positive perceptions around the use and integration of learning analytics for mental health reflect the need to align learning analytics practices with mental health strategic priorities as part of a whole-university and data-driven approach to mental health [4]. Second, students’ ethical concerns regarding learning analytics adoption, particularly around transparency, consent, and privacy, highlight the importance of involving students in their development and implementation, echoing previous policy calls [15,16,27-30]. Universities should, therefore, develop clear institutional policies and protocols coproduced with students regarding the development and implementation of learning analytics. Third, institutional learning analytics policies should align with national higher-education–wide guidelines to ensure sector-wide consistency. A coordinated government strategy on student mental health is needed to align institutional practices with broader policy goals. Ultimately, while learning analytics offer strong potential to enhance student support and service delivery, their success relies on meaningful student involvement, institutional accountability, and coherent policy at both institutional and national levels.

### Conclusion

This was the first study to explore students’ perceptions of using learning analytics to support students’ mental health and well-being. Our findings suggest that students perceive value in utilizing learning analytics to enhance mental health and well-being in higher education, as well as their integration with existing university services, which holds significant importance for UK policy. However, considering the ethical concerns raised by students, this study also highlights the need to inform students about learning analytics–based systems being developed or implemented at their institution, echoing previous calls for student engagement in the decision-making process regarding learning analytics. Ensuring learning analytics systems meet students’ needs and expectations is essential to avoid resistance to learning analytics adoption and empower students to make decisions regarding their welfare and educational experience. Overall, learning analytics holds promises for its integration within university support, but not without the involvement of students.

## Supplementary material

10.2196/70327 Multimedia Appendix 1 Supplementary materials include the interview topic guide, figures of thematic map development, documentation of patient and public involvement activities, a table of full participant demographics, and a reflexivity statement.

10.2196/70327 Checklist 1 Consolidated Criteria for Reporting Qualitative Research (COREQ) checklist.

## Abbreviations

AI: artificial intelligence
COREQ: Consolidated Criteria for Reporting Qualitative Research
PPI: patient and public involvement
SAG: student advisory group
STEM: science, technology, engineering, and mathematics
