# Supplementary material for: Students’ Perceptions of Learning Analytics for Mental Health Support: Qualitative Study
Source: JMIR Form Res. 2025 Aug 1;9:e70327. doi: 10.2196/70327 (PMC12316473; doi:10.2196/70327)
Supplement: Multimedia Appendix 1 [file formative-v9-e70327-s001.doc]

Appendix S1: Semistructured interview guide.

ICREC Topic Guide (Version 1, 24/05/23)

**Indicative questions for semi-structured interviews:**“Students’ perceptions of using learning analytics to support university students’ mental health: a qualitative study”

1. **Opening**: Thank you for agreeing to participate in this research. Please state for the audio and video recording that you have had an opportunity to read the Research Information Sheet and that you have consented to take part in this study.

Hello, my name is Aglaia, and I am interested in exploring students’ views on using learning analytics to support students’ mental health at university for my research project as part of my Master in Public Health at Imperial College London.

Note: give background to the reason for am carrying the project. Provide some context to learning analytics in education and their potential use for well-being purposes at university.

1. **Introductory questions** *(helping people feel comfortable and at ease)* 5-10 minutes

- *Example questions can include: what they’ve done today so far or how they are in general.*
- *Specify that this is a safe space in which to talk openly about their experiences and recap on the importance of confidentiality, withdrawal and stopping if at any point they wish to have a break.*

Topic 1: Existing Mental Health Support at University

1. What are your thoughts regarding the existing mental health support at university? Prompts:
   *Is it sufficient? Why/why not?*
2. What do you think are the problems with existing mental health support at university, if any?
3. If you believe there are some problems with existing mental health support at university, what do you think could be done to address these problems?

Topic 2: Learning Analytics and Well-being

1. What kind of data do you think your university holds regarding your learning?
   (*Provide* *an accurate definition of learning analytics to refer in following questions)*

Learning analytics refers to “the measurement, collection, analysis, and reporting of data about learners and their contexts, for the purposes of understanding and optimising learning and the environments in which it occurs” (Siemens 2012).

Data used in learning analytics applications comes from existing university systems, student information systems, VLEs, library data, and attendance monitoring, all of which are behavioural data.

1. Please can you describe your views on using learning analytics to support university students’ well-being?
2. How do you think this approach could be used to support university students’ well-being? Probing questions:
   *Can you give me some examples?*
3. What are your views on using learning analytics for well-being monitoring and early intervention?

Topic 3: Facilitators and Barriers

1. What could be done to support the use of learning analytics for well-being purposes?
2. Do you see any issues arising around the use of learning analytics for well-being purposes? Prompts:
   a. Consent
   b. Privacy
   c. Equity
   d. Ownership
   e. Academic Impact
3. Tell me about what impact, if any, you believe this would have on academic outcomes.

Topic 4: Integration with Existing Services

1. How do you think this new approach to support students’ well-being could affect student mental health at university? Probing questions:
   *What sort of impact do you think this will have? Why?*
2. What are your views on integrating the use of learning analytics into current mental health support available at university?
3. Do you have any recommendations on how this could be achieved?

Topic 5: Additional Comments

1. Is there anything else you would like to discuss that we have not already talked about?

- Thanks, and debrief (including options for support if necessary)

Figure S1: Thematic map development.

**
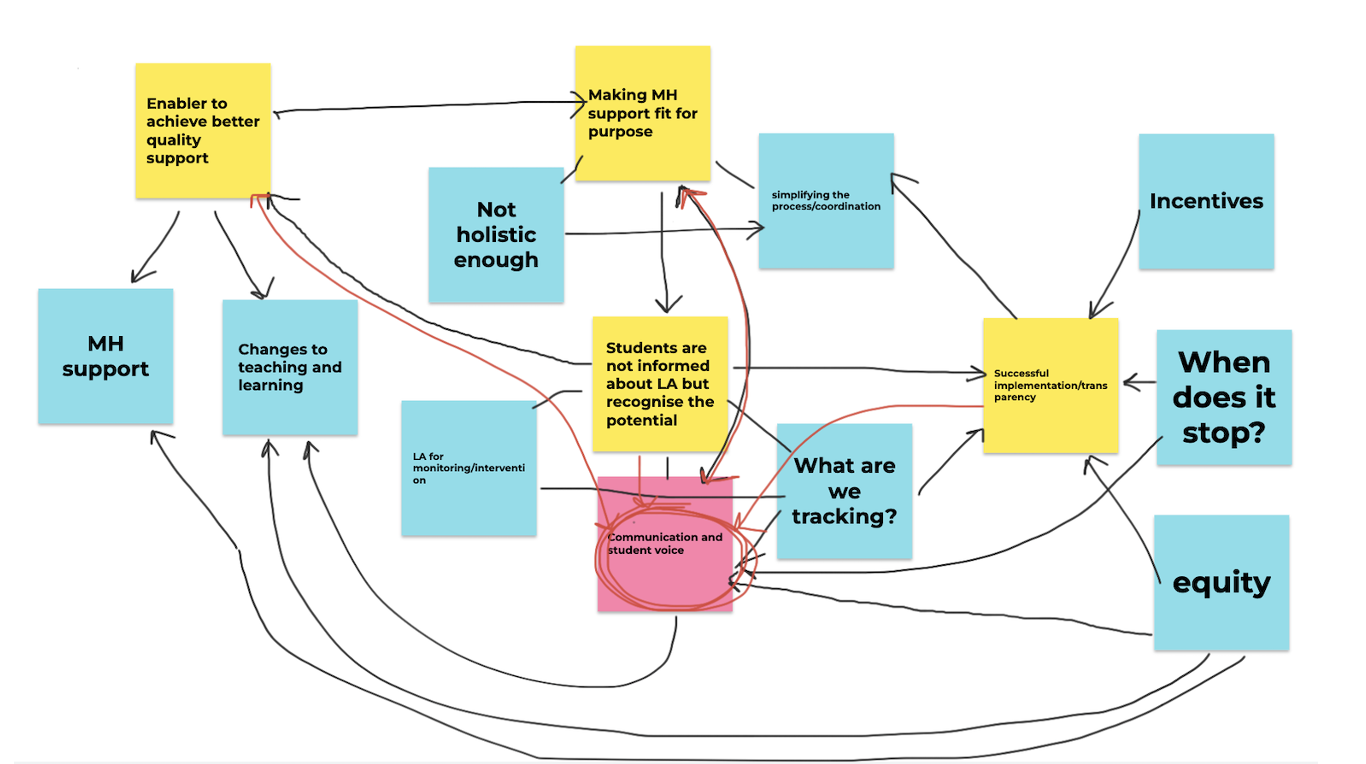
**

Version 1: meeting with PPI group on July 14^th^ 2023


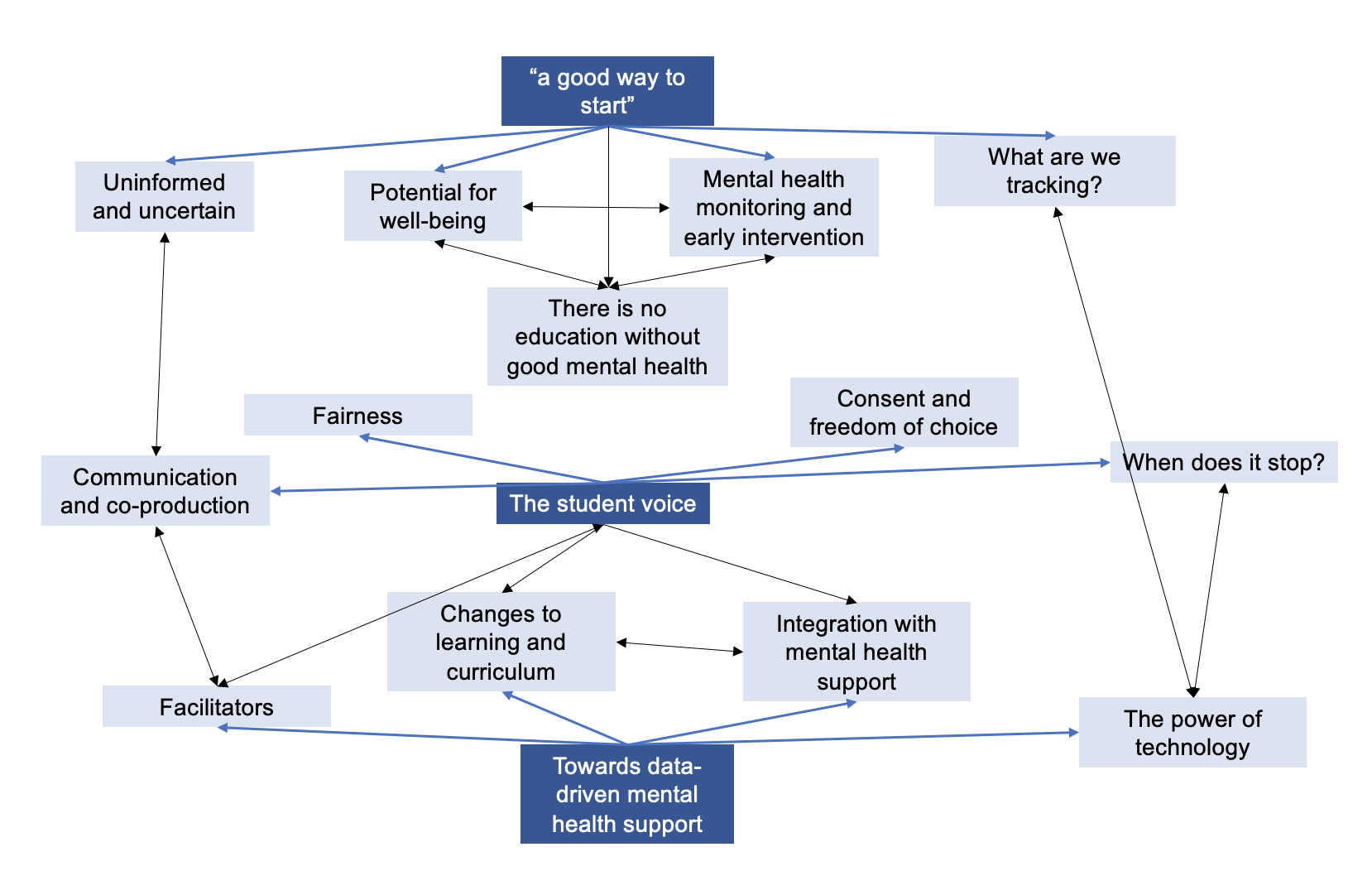


Version 2: meeting with PPI group on August 2^nd^ 2023

Table S1: GRIPP2 short form.

| **Section and topic** | **Item** | **Reported on page No** |
| --- | --- | --- |
| 1. Aim | Report the aim of PPI in the study | Page 3 |
| 1. Methods | Provide a clear description of the methods used for PPI in the study | Page 3 |
| 1. Study Results | Outcomes – Report the results of PPI in the study, including both positive and negative outcomes | Page 4 |
| 1. Discussion and conclusions | Outcomes – Comment on the extent to which PPI influences the study overall. Describe positive and negative effects. | Page 8 |
| 1. Reflections/critical perspective | Comment critically on the study, reflecting on the things that went well and those that did not, so others can learn from this experience | Page 8 Appendix S1 |


Table S2: Participants full demographics.

| **Participant ID** | **Age** | **Gender** | **Sexual Orientation** | **Ethnicity** | **University** | **Qualification** | **Year of study** | **Fee status** | **Degree** |
| --- | --- | --- | --- | --- | --- | --- | --- | --- | --- |
| 1 | 20 | F | Heterosexual | White | University College London | Undergraduate | 2 | International | BSc Biochemistry |
| 2 | 23 | F | Heterosexual | Asian - Chinese | University College London | Undergraduate | 3 | International | MSci Biochemistry |
| 3 | 20 | M | Heterosexual | Asian -Chinese | Warwick | Undergraduate | 2 | International | BSc Mathematics |
| 4 | 23 | F | Homosexual | Asian - Indian | Imperial College London | Undergraduate | 3 | Home | Medicine MBBS |
| 5 | 19 | M | Heterosexual | Asian – Hong Kong | Imperial College London | Undergraduate | 1 | International | BSc Medical Biosciences |
| 6 | 22 | F | Homosexual | White | Imperial College London | Postgraduate | 4 | Home | MSci Physics |
| 7 | 21 | F | Heterosexual | White | University College London | Undergraduate | 3 | Home | BSc Human Sciences |
| 8 | 24 | M | Heterosexual | Other - Persian | Imperial College London | Postgraduate/PhD | 2 | International | PhD Department of Bioengineering |
| 9 | 22 | M | Heterosexual | Mixed White/Asian | Imperial College London | Postgraduate/MSc | 1 | Home | MSc Translational Neuroscience |
| 10 | 22 | F | Heterosexual | Black - African | Imperial College London | Undergraduate | 3 | International | BSc Medical Biosciences |
| 11 | 20 | M | Heterosexual | Mixed White/Asian | Imperial College London | Undergraduate | 2 | Home | BSc Biochemistry |
| 12 | 19 | F | Heterosexual | Asian - Chinese | Imperial College London | Undergraduate | 2 | International | BSc Biological Sciences |
| 13 | 21 | F | Heterosexual | Mixed White/Asian | King’s College London | Undergraduate | 1 | Home | Medicine MBBS |
| 14 | 23 | F | Heterosexual | White | Imperial College London | Postgraduate/MSc | 1 | Home | MSc Translational Neuroscience |
| 15 | 23 | F | Heterosexual | White | Imperial College London | Undergraduate | 6 | Home | Medicine MBBS |

*Asian or Asian British: Indian, Pakistani, Bangladeshi, Chinese, Any other Asian background

Black, Black British, Caribbean or African: Caribbean, African, any other Black, Black British or Caribbean background

Mixed or multiple ethnic groups: White and Black Caribbean, White and Black African, White and Asian, Any other Mixed or multiple ethnic group

White: English, Welsh, Scottish, Northern Irish, British, Irish, Gypsy or Irish Traveller, Roma, any other White background

Other ethnic group: Arab, Any other ethnic group

**Home Fee Status: students ordinarily resident and or settled in the UK

International Fee Status: Overseas or international students not subject to home fee status

Appendix S2: Reflexivity Statement.

I am a 27-year-old student enrolled in a PhD in the Division of Psychiatry at Imperial College London. I identify as a white heterosexual female, and I was born in Italy, where I attended public education. I am, therefore, aware of the issues encountered by international students when transitioning into university in the UK. I completed an MSc in Translational Neuroscience and a Master’s in Public Health at Imperial College London, and a BSc in Biomedical Sciences at University College London. Having attended two large and prestigious institutions in London, I have been exposed to an academically challenging and diverse environment. I have both clinical and non-clinical research experience, and my PhD explores the effectiveness of AI-informed mobile behavioural interventions to support adolescents in schools. Thus, when conducting research, I can draw from my clinical understanding of mental health and the use of data-driven tools to support young people’s mental health.

My interest in mental health research is also grounded in my lived experience of mental ill-health. In 2018, I was diagnosed with bipolar II disorder, and, since then, I have suffered from the personal and academic toll of my illness. I am, therefore, likely to find salient experiences that match my own. During my BSc, I sought academic and mental health support from the university and interrupted my studies for one year. I struggled to navigate the university support system, and what I was offered often did not meet my needs. I thus identify with a general feeling of discontent with the current university mental health support, and I feel compelled to call attention to these issues and strive to address them. I believe in adopting a student-centric approach to meet student needs and involving them in the decision-making process.

I have been a very active member of the Imperial College Union, and from April 2022 until October 2024, I was the elected Mental Health Officer. Part of my role was to represent student mental health needs and improve the experience and support provisions for students at Imperial College London. I have contributed to developing the university’s first institutional mental health strategy, aiming to create a supportive environment where students can thrive and belong. As part of this work, I joined the Imperial College London Learning Analytics Steering Group, an advisory board directing the vision and development of the Learning Analytics Project. The motivation for this study stems from my involvement in this project as a student advisor and reflects my continuous commitment to student advocacy and representation.
